# TaILOR: a randomised trial to compare the clinical and cost-effectiveness of a patient-initiated follow-up (PIFU) strategy compared to standard care pathways in people with inflammatory arthritis: a study protocol

**DOI:** 10.1186/s13063-026-09868-0

**Published:** 2026-07-04

**Authors:** Gretchen Brewer, Victoria Harris, Christopher Mwema, Abhishek Abhishek, Ailsa Bosworth, Christine Bundy, Clare Clark, Elizabeth Conroy, Helen Dakin, Emma Dures, Joe Eddison, Anne Francis, James Galloway, Alice Jennings, Helen Lall, Elizabeth MacPhie, Sam Norton, Mark Perry, Jane Raleigh, Mel Brooke Turfrey, Laura Coates

**Affiliations:** 1 University of Oxford, Oxford, UK; 2 University of Nottingham, Nottingham, UK; 3 National Rheumatoid Arthritis Society, Berkshire, UK; 4 Cardiff University, Cardiff, UK; 5 National Axial Spondyloarthritis Society (NASS), London, UK; 6 University of the West of England, Bristol, UK; 7 King’s College London, London, UK; 8 London, UK; 9 Lancashire and South Cumbria NHS Foundation Trust, Preston, UK; 10 University Hospitals Plymouth NHS Trust, Plymouth, UK; 11 NHS England, London, UK; 12 BritPACT Patient Group, Bath, UK

**Keywords:** Patient Initiated Follow up (PIFU), Patient-led follow-up, Patient-triggered follow-up, Supported self-managed follow-up, Inflammatory arthritis, Standard care, Musculoskeletal, Quality of life

## Abstract

**Background:**

Inflammatory arthritis comprises lifelong conditions, such as rheumatoid arthritis, axial spondyloarthritis and psoriatic arthritis that require long-term treatment and regular monitoring. People with inflammatory arthritis usually require long-term treatment with immune-suppressing medications and are typically reviewed in outpatient clinics every 6–12 months, accounting for 1.3 million appointments/year in the UK. The National Health Service (NHS) is implementing a move to patient-initiated follow-up (PIFU) where patients request appointments as required. To date, there are small studies of PIFU in rheumatoid arthritis and none in other arthritis. Research is required to configure services to optimise outcomes, inform patients and clinicians of the effectiveness and safety of PIFU and ensure that suitable patients are selected for this pathway.

**Methods:**

TaILOR is a two-arm, pragmatic, parallel group, superiority, randomised controlled clinical study, with an embedded qualitative study conducted across 32 secondary care NHS sites in the UK. Eligible participants must be 18 years or older, with stable inflammatory arthritis, and diagnosed for at least 2 years. In addition, they need to be suitable for PIFU based on NHS England guidance and in the opinion of their usual care team; 438 participants will be randomised in a 1:1 ratio to either PIFU, with a fixed 24-month safety net appointment, or standard care, with 6–12 monthly follow-up visits. The trial includes an internal recruitment feasibility pilot, as well as health economics and qualitative analyses. The primary outcome will assess the effectiveness of PIFU compared to standard care on musculoskeletal quality of life at 24 months. Secondary outcomes include quality of life, incremental costs, cost-effectiveness, treatment escalation, disease activity, flares, patient confidence in interactions with their clinical team (patient perceived efficacy in patient-physician interactions), depression and acceptability of PIFU (via qualitative interviews).

**Discussion:**

As a national multi-centre study, this will provide the definitive evaluation of PIFU for inflammatory arthritis. Qualitative analysis will generate insights into individual and contextual factors affecting the acceptability of PIFU, identify variation across sites or between patients and provide information on how this can be addressed.

**Trial registration:**

ISRCTN ISRCN10480648. Registered on 17 January 2025.

Protocol version {2}: Version 3.0 date 04 Dec 2025.

**Supplementary Information:**

The online version contains supplementary material available at 10.1186/s13063-026-09868-0.

## Introduction

### Background and rationale {9a}

Inflammatory arthritis comprises long-term conditions with an inflammatory process in the joints, and includes rheumatoid arthritis (RA), axial spondylitis (AxSpa) and psoriatic arthritis (PsA). The estimated combined prevalence in the United Kingdom (UK) is >1.5% [1]. Joint inflammation causes pain and functional impairment, irreversible joint damage, and disability. These diseases require lifelong management by hospital rheumatology multi-disciplinary teams (MDTs) using both immune-suppressing medicines (called disease-modifying anti-rheumatic drugs (DMARDs)) and non-pharmacological treatments. Even while on treatment to control inflammation, prevent joint damage, and improve symptoms and function, inflammatory arthritis often causes unpredictable fluctuations in disease activity, called flares.

Traditionally, patients with inflammatory arthritis have been offered periodic, routine, outpatient follow-up appointments, typically 6–12 monthly. This accounts for 1.3 million appointments/year. During these appointments the patient describes symptoms and the clinician performs clinical assessments, i.e. blood and/or imaging tests to check for inflammation and adjust treatment as appropriate. However, patients may be well at their visit time and not require a treatment change or advice about other aspects of their disease. In a focus group held prior to study design, a patient told us, “Often you feel very good when you go in… but really, you need to see somebody when you don’t feel so good.” This model of service delivery may also make it difficult for both patients and clinical teams to provide timely advice or review when required at times other than routine appointments.

In 2019, The NHS Long Term Plan [2] highlighted the challenges in relation to traditional outpatient delivery models. It argued for people to have more control over their own health and more personalised care when they need it. Instead of attending routine, intermittent 6–12 monthly follow-up appointments, patients (or their carers) on patient-initiated follow-up (PIFU) pathways initiate contact with their treating service and, if required, arrange an appointment [3]. There is an urgent imperative to review outpatient services now given waiting times and the large backlog in rheumatology. The 2022/23 NHS Priorities and Operational Planning Guidance [4] requested that hospitals expand the uptake of PIFU to all major outpatient specialties, moving or discharging 5% of outpatient attendances to PIFU pathways by March 2023. This rollout allows for trials like TaILOR to evaluate the effectiveness of PIFU in rheumatology, informing clinical practice and outpatient service delivery models for the future. Recent health policy documents including “Reforming elective care for patients” and “Fit for the future – The 10-year health plan for England” support the approach to reform elective care aligned with the “analogue to digital” shift where patients will be able to communicate with healthcare teams via the NHS application [5, 6].

Guidance on implementing PIFU in adult rheumatology services has been developed and published by NHS England (NHSE) [7]. PIFU, with appropriate shared decision-making, may offer an opportunity to empower patients, supporting self-management and a sense of control over healthcare decisions. It may improve patients’ experience of care and enable clinicians to focus their attention on those whose clinical need is greatest. However, there are concerns from patients and clinicians, raised in Patient and Public Involvement (PPI) work developing the study, that PIFU should not compromise patients’ short-term or long-term outcomes, nor result in increased health inequalities and that PIFU should be tailored to the individual patient and service concerned.

There are currently no studies ongoing assessing the effectiveness and cost-effectiveness of PIFU in the UK. A Cochrane review in 2020, addressing research across all long-term conditions, included data on 3402 patients, of whom 909 had inflammatory arthritis [8]. These four PIFU studies in rheumatology all had indicators of bias [9–12]. Three studies were single-centre and one studied two centres. All studies included only patients with RA and only one was conducted in the UK [9] well before the widespread use of biologic drugs that have had paradigm shifting effects on patient outcomes. We performed a meta-analysis of these studies, which showed that studies are inconclusive with respect to indicating whether PIFU is beneficial or harmful. As expected, PIFU is associated with fewer visits, consistent with ~1 fewer visit per-patient per-year, but a higher number of helpline calls [13]. It is uncertain whether these outcomes are generalisable to other centres, with different clinical pathways and to other conditions. A multi-centre randomised controlled trial is needed to establish whether PIFU is superior to standard care for people with inflammatory arthritis, and which patients benefit the most.

### Explanation for the choice of comparator {9b}

As TaILOR was designed to be a pragmatic study, the comparator (standard care) was chosen to reflect the follow-up care pathways available to patients in secondary care in the NHS.

## Objectives {10}

The aim of the study is to assess the effectiveness and cost-effectiveness of PIFU compared to standard care in terms of musculoskeletal QoL outcomes for patients with inflammatory arthritis. We will also undertake qualitative work to gain insight into the barriers, facilitators and acceptability of PIFU from the perspective of patients and NHS professionals.

This study is part of a programme of work to establish whether and/or in which circumstances PIFU is superior to standard care for people with inflammatory arthritis. The data arising from this study will be used, along with data from a separate observation study on routine data undertaken as part of this programme, to investigate factors associated with better PIFU outcomes and to enable extrapolation of the trial results to the wider UK population.

## Methods: patient and public involvement, and trial design

### Patient and public involvement {11}

#### PPI in study design and protocol development

From conception, this study has been developed in conjunction with PPI including patients and representatives from the National Rheumatoid Arthritis Society (NRAS), National Axial Spondyloarthritis Society (NASS) and the British PsA Consortium (BritPACT). A patient survey of nearly 1000 respondents in May 2023 identified highly variable confidence with a PIFU approach, but the vast majority (67%) said they would be willing to take part and be randomised to PIFU or standard care in a randomised controlled trial (unpublished data).

We have 4 patient partners (of which 3 are co-applicants) to provide diversity of views and experiences, ensure that they do not feel isolated, to account for different types of IA, and to allow for any absences due to problems with their arthritis. The 3 PPI co-applicants for the study are patients and representatives of relevant charity organisations (NRAS, NASS and BritPACT) and have extensive PPI experience. All of the patient research partners have lived experience of inflammatory arthritis and represent different diagnoses within this group of conditions and varying experiences with NHS treatments and care. Two of the three co-apps are experienced patient partners who play an active role in local and national groups.

Very early in the concept, the patient partners supported the idea of a study investigating the effectiveness of a PIFU approach and how to optimise this. They recognise the frustration of pre-booked appointments which may not fall at times of clinical need, but wanted to ensure that care is optimally designed to ensure that people are not disadvantaged or suffer joint damage without routine review and have access to care when required. They were also keen to support patients in accessing other care such as physiotherapy, hand/occupational therapy. They supported the pragmatic design of the study as they were keen for the study results to be generalisable across the UK and wanted to minimise burden on participants.

To allay their concerns about the quality of a PIFU pathway, in 2023 investigators from the TAILOR study ran a project to co-develop a PIFU manual for all hospital sites with support of the British Society for Rheumatology providing core requirements of the PIFU approach. The same patient partners were involved in this project providing continuity. This project developed patient educational materials that can be utilised by all sites and promoted by relevant patient organisations.

Selection of the primary and secondary outcomes was discussed with the patient partners. When we asked the patient partners in an early stage of development, the key metric they wanted was of “good care” where patients are satisfied with the quality of their care and disease management. They were keen to use a quality-of-life measure as the primary outcome to reflect this holistic outcome and felt that the Musculoskeletal Health Questionnaire (MSK-HQ) would be meaningful to patients and be applicable across diseases. They were also involved in the selection of secondary outcomes including measures of disease activity (e.g. joint counts), disease impact, patient satisfaction and patient activation.

#### PPI in development of participant information

The patient co-applicants were involved in the development and review of the patient information for the study. The documents reviewed include the study infographic, invitation letters, patient information sheets for both the main study and the qualitative study, a survey for patients who decline to take part in the study, topic guides for the qualitative study, patient facing questionnaires, questionnaire covering letters and advertising material.

#### PPI during the study, including analysis and dissemination of results

Patients and the public will be actively involved throughout the study. During the study, we will continue the relationship with the patient co-applicants. All co-applicants are invited to attend the monthly Co-Applicant meeting and one co-applicant is part of the Trial Steering Committee (TSC). The value of patients in previously conducted trials is recognised by the supporting Clinical Trial Unit (CTU).

Individuals who agree to contribute to the project management have met with the CTU to gain understanding of their previous involvement in research (if any). Appropriate training was offered, including the induction pack for PPI contributors on Trial Oversight Committees. The lead applicant has worked in strong collaboration with patient partners within BritPACT, GRAPPA (Group for Research and Assessment of Psoriasis and Psoriatic Arthritis), OMERACT (Advancing Outcome Measurement in Rheumatology) and individual studies and also leads the PPI group OPEN ARMS (Oxford Patient Engagement Network for Arthritis and Musculoskeletal Conditions) supporting PPI across the NDORMS (Nuffield Department of Orthopaedics, Rheumatology and Musculoskeletal Sciences) division at University of Oxford. Training and support will be offered through OPEN ARMS. A specific PPI co-ordinator will provide a single point of contact when required.

As well as providing a patient perspective on all aspects of the study and its execution, the patient research partners have helped with decisions on the best ways of recruiting and study burden. In addition, with appropriate permission, short videos of study participants may be recorded, providing their experience of the study.

Patient partners will assist in the interpretation of overall grant findings and how these may be communicated to the general public and positioned for further investigation. Where appropriate, patient advisors will be co-authors on publications. As a NIHR funded project, the required reports will be produced; however, we will work with our PPI collaborators to ensure any plain English parts of the monograph are phrased appropriately, and we intend to produce an infographic if possible, to explain the findings.

### Trial design {12}

The TaILOR study is a multi-centre, two arm, parallel group, superiority, randomised controlled clinical study, with an embedded qualitative study and within-trial cost-utility analysis.

## Methods: participants, interventions and outcomes

### Trial setting {13}

The study will recruit 438 patients (219 in each of two arms) with stable inflammatory arthritis from 32 sites, typically secondary-care NHS hospitals, community-based NHS rheumatology clinics in the UK. A list of participating sites can be found on the ISRCTN registry [14].

### Characteristics of the people who are needed for the trial

**Table:** 

Characteristic	The people we would expect to see included
Age	18 years and older
Sex	Male, female
Gender	Men and women
Race, ethnicity and ancestry	Participants of any race/ethnicity can be included Asian, Asian British or Asian Welsh Black, Black British, Black Welsh, Caribbean or African Mixed or multiple ethnic groups White Other ethnic group
Socioeconomic status	Employment Status (Employed, full time; Employed, part time; Self-employed; Care giver; Out of work and looking for work; Out of work but not currently looking for work; Unable to work; Homemaker; Student; Military; Retired); Prefer not to say Education Level: (No formal education; Primary education (educated to age 11 or before); Secondary education (educated to age 18 or before); Higher education (e.g. Diploma, HNC); University education); Prefer not to say Income: (£0-£30,000; £31,000- £60,000; £61,000- £90,000; £91,000- £120,000; £120,000 + ; Prefer not to say)
Geographic location	Participants living in England, Scotland, Wales and Northern Ireland are being recruited from centres selected to represent a mix of urban and rural areas. The study coordination centre is located in England
Other characteristics relevant to the trial	NA

### Eligibility criteria for participants {14a}

A patient will be eligible for inclusion in this study if all of the following criteria apply:

- Aged 18 years or over.
- Diagnosis of inflammatory arthritis (RA, PsA, AxSpA, undifferentiated arthritis) for at least 2 years.
- Stable disease: defined as a level of disease control that the rheumatology team feels is suitable for PIFU; on the same conventional, targeted synthetic or biologic DMARD(s), or no treatment, for at least the previous 3 months; and with no escalation in therapy planned.
- Able to contact the Rheumatology team when required.
- Suitable for PIFU in the opinion of their rheumatology team.
- Willing and able to give consent and adhere to study procedures.

A patient will not be eligible for the study if any of the following apply: 

- Currently or previously on PIFU for inflammatory arthritis.
- Safeguarding/consent/capacity concerns (using General Medical Council guidance).
- Health literacy concerns from the treating clinician related to inflammatory arthritis.
- Women who are pregnant or planning to start a family^1^.
- Currently undergoing radiotherapy, immunotherapy or chemotherapy for malignancy.
- Patients on end-of-life care pathways.

### Eligibility criteria for sites and those delivering interventions {14b}

Sites are eligible to participate in the study if they have a PIFU pathway for inflammatory arthritis in place at the start of recruitment.

### Who will take informed consent? {32a}

Informed consent from each participant will be sought and collected by a member of the site research team before they undergo any study-related procedures or interventions related to the study. A member of the site research team will provide a copy of the Participant Information Sheet (PIS) and answer any questions that the potential participant has concerning the study. If the potential participant agrees to give consent, both the participant and the Investigator (or authorised designee) must personally sign and date the current approved version of the Informed Consent Form (ICF). The ICF will usually be offered to participants in clinic as an electronic form on a tablet device (with the consent form being filled in directly on the Research Electronic Data Capture (REDCap) system); however, paper consent forms will also be made available for use in situations where electronic consent is not possible or suitable.

### Additional consent provisions for collection and use of participant data and biological specimens {32b}

Eligible individuals who agree to participate in the study will also be informed about the qualitative elements of this study and will be asked if they are willing to give consent to be potentially contacted about participating in a qualitative interview. Participation in this element of the research is voluntary and refusal to participate will not affect their inclusion in the clinical study.

Those participants who indicate they are willing to be contacted about participating in a qualitative interview and are selected to be interviewed will be contacted directly by the qualitative team and provided with further information about participating in up to two interviews. Ahead of the first interview, the qualitative team will provide the qualitative study information sheet and consent form to the participant, usually via email. If the participant is willing to take part in the interview(s), the participant will be asked to complete and return a consent form to the qualitative team via email. However, if a completed consent has not been received by the time of the interview, consent to be interviewed may also be obtained verbally. The research team will first talk the participant through the interview process, then go through the consent statements clarifying that the participant understands and agrees with each statement. The researcher will complete a remote consent form on behalf of the participant, sign the form, scan and email a copy to the participant for their records, retaining a copy in a secure location. Consent will be obtained prior to starting an interview and before any data collection.

If the participant is invited for a second interview, the researcher will confirm ongoing consent with the participant ahead of the interview. The participant can withdraw consent from the interview(s) at any time.

## Intervention and comparator

### Intervention and comparator description {15a}

Participants randomised to PIFU will access PIFU when they require specialist rheumatology or MDT input. They will access PIFU as per the pathway of their hospital/care site. Localised information on how and when to access PIFU if they require specialist rheumatology input will be provided to participants randomised to PIFU by their rheumatology team. This includes published patient information disseminated by the British Society for Rheumatology.

A month 24 appointment — a PIFU safety net appointment — will be booked for routine follow-up.

All study sites will be supplied with current NHSE guidance on PIFU and resources developed in 2023–2024 by members of the research team in collaboration with the British Society for Rheumatology (BSR). These resources (listed below) can be found on the BSR website [15].

- The standard PIFU manual which will be utilised to support sites on patient selection, patient education and use of digital Patient Reported Outcomes (PROs) for monitoring.
- An explanatory video about PIFU for patients which is available with subtitles in English, Welsh, Polish, Urdu, Punjabi, Romanian and Cantonese.
- A FAQ document about PIFU for patients that can be amended to reflect the PIFU pathway of their hospital/site.

As PIFU rollout varies across rheumatology centres, site-specific PIFU information will be collected at the beginning and the end of the trial to be able to describe PIFU practices in rheumatology nationwide.

The comparison chosen was “standard care” as provided in UK rheumatology departments, despite a recognition that this will vary between sites. Participants randomised to standard care will have booked appointments at 6-to-12-month intervals over the 2-year study period in accordance with local practice. Patients allocated to standard care must not be moved to PIFU as part of the NHS/local Trust’s PIFU rollout. Clinical data routinely collected during the month 24 standard care appointment will be collected for study purposes.

### Criteria for discontinuing or modifying allocated intervention/comparator {15b}

Participants allocated to PIFU may be switched back to standard follow-up either at the discretion of their clinical care team if PIFU is no longer felt to be suitable for them or upon patient request. This will be recorded but primary analysis will be based on intention to treat (ITT) (i.e. a treatment policy estimand). Additional secondary analysis looking at other estimands based on adherence will be detailed in the statistical analysis plan (SAP).

### Strategies to improve adherence to intervention/comparator {15c}

There are two time points during the follow-up period where adherence to the intervention will be recorded. At 6–8 weeks and 11 months after randomisation, the site research team are to review the participant’s medical records to ensure that the participant has been allocated to the appropriate follow-up appointment type i.e. those allocated to PIFU have not been booked for a routine visit in 6–12 months. Should any errors be found, a member of the site research team will contact the relevant local team/person to correct the error.

A final adherence check will be done at the 24 month visit where the site research team are to review the participant’s medical records to ensure that the participant has been allocated to the appropriate intervention. If an error has been found, this will be recorded in the Case Report Form (CRF).

### Concomitant care permitted or prohibited during the trial {15d}

There are no concomitant care or interventions that are prohibited during the trial.

### Ancillary and post-trial care {34}

At the end of trial participation, follow-up in rheumatology will continue following standard practice of that centre. Participants will discuss with their clinical care team whether they wish to continue with standard follow-up or PIFU care. Insurance from the study sponsor (University of Oxford) covers any harm suffered by participants related to study participation.

### Outcomes {16}

A full list of primary, secondary and exploratory outcomes is given in Table 1. The primary outcome is the MSK-HQ at 24 months, with additional assessments throughout the 24-month study through remote data collection in both groups every 6 months via email, post or telephone. We feel that the most important outcome is the overall impact of PIFU on patients.

**Table 1:** Primary and secondary outcome measures

Primary objective	Outcome measure	Time point(s) of evaluation of this outcome measure (if applicable)
To assess the effectiveness of PIFU compared to standard care on musculoskeletal quality of life at 24 months	MSK-HQ score	Baseline, months 6, 12, 18 and 24
**Secondary objectives**	**Outcome measure(s)**	**Time point(s) of evaluation of this outcome measure (if applicable)**
To assess the effectiveness of PIFU compared to standard care on musculoskeletal quality of life over 24 months	MSK-HQ score	Baseline, months 6, 12, 18, and 24
To assess the effectiveness of PIFU compared to standard care on musculoskeletal quality of life at 6, 12 and 18 months	MSK-HQ score	Baseline, months 6, 12, 18 and 24
To assess the effectiveness of PIFU compared to standard care on overall health related quality of life	EuroQol 5 Dimensions 5 Level Version (EQ-5D-5L) and EuroQol Visual Analogue Scale (EQ-VAS) scores	Baseline, months 12 and 24
To assess the incremental cost of PIFU compared to standard care	Incremental cost	Baseline, months 6, 12, 18 and 24
		12 months prior to Baseline through month 24
To assess the cost-effectiveness of PIFU compared to standard care	Cost per quality adjusted life year (QALY) gained	24-month time horizon
To compare the effect of PIFU versus standard care on progression from no treatment to first line DMARD	Proportion of patients starting a first line DMARD during the study	24 months
To compare the effect of PIFU versus standard care on progression from conventional drugs to biologic therapies	Proportion of patients starting a first biologic during the study	24 months
To compare the effect of PIFU versus standard care on disease activity	Clinical disease activity index (CDAI) and disease activity score (DAS28-CRP) for Rheumatoid Arthritis (RA) Ankylosing spondylitis disease activity score (ASDAS) for Axial Spondyloarthritis (AxSpA) Disease activity in psoriatic arthritis (DAPSA) for Psoriatic Arthritis (PsA)	Baseline and 24 months
To compare the effect of PIFU versus standard care on disease activity	Number of flares	Baseline and 24 months
To compare the effect of PIFU versus standard care on patient efficacy	Perceived efficacy in patient-physician interactions (PEPPI) score	Baseline and 24 months
To compare the effect of PIFU versus standard care on depression	Mental health using Patient Health Questionnaire-4 (PHQ-4)score	Baseline and 24 months
To evaluate the acceptability of PIFU to patients and health professionals/service providers	Semi-structured interviews	Weeks 2–12 and Months 16–24
		Once site has been open to recruitment for at least 12 months
**Exploratory objectives**	**Outcome measure**	**Time point(s) of evaluation of this outcome measure (if applicable)**
To explore potential risk factors, and their association with PIFU outcomes	MSK-HQ score	Baseline, months 6, 12, 18 and 24
	Disease activity score	24 months
To explore the reasons patients declined to participate in the TaILOR study	Online and/or paper brief survey designed by the team	During recruitment

We need to rely on a PRO that can be completed remotely as we believe that there is a risk that bringing patients in for a study visit during the two-year follow-up, may contaminate the PIFU approach, which is an issue with previous trials. Many PROs, such as the health assessment questionnaire (HAQ) were rejected by the patient partners as they were felt to be too outdated, only measuring significant levels of physical impairment rather than more subtle impacts of the disease.

The MSK-HQ is a patient-reported outcome assessing musculoskeletal Health Related Quality of Life (QoL) that has been validated in patients with inflammatory arthritis. The MSK-HQ was developed in 2016 by Arthritis Research UK to capture constructs specific to musculoskeletal health but designed for use across a range of rheumatological conditions and settings [16]. It incorporates measures of symptoms, e.g. pain and disease impact and was developed with significant patient input. Scores range from 0 to 56 with higher scores representing better musculoskeletal health. The MSK-HQ includes domains key to patients’ perception of musculoskeletal health, correlates highly with other PROs in inflammatory arthritis and has robust psychometric properties [17].

### Harms {17}

As PIFU is considered to be standard care within the NHS, safety reporting is not applicable for this study. Patient reported flare information (total number of flares as well as the number that required follow-up with a medical provider and steroid treatment) will be collected every 6 months and summarised and reviewed by the DMC.

Although no conventional biomedical adverse events are anticipated, we recognise the potential for inequitable distribution of benefits or unintended social harms associated with the intervention. To mitigate this risk, the study has been designed with a strong focus on inclusivity, including recruitment across diverse populations and settings, flexible participation options, multilingual materials, and ongoing monitoring of ethnicity and deprivation data. Recruitment patterns will be reviewed by the Trial Management Group (TMG) to identify under-representation of particular groups and implement targeted actions where required. These measures will also allow exploration of whether intervention uptake, engagement, or outcomes differ across population subgroups.

### Participant timeline {18}

**Fig. 1:**
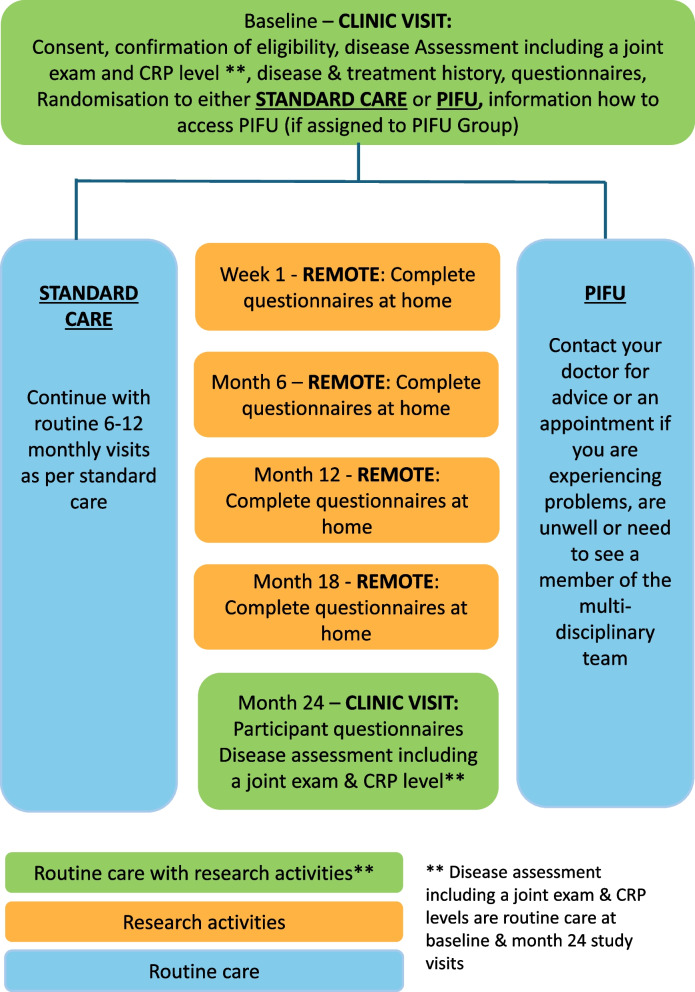
Participant timeline

Regardless of the study arm, there are only two timepoints (baseline and month 24) at which hospital attendance is required for the purpose of the study. These are both routine care visits at which the relevant disease activity score will be determined (as part of routine care) or extracted from the participant’s medical notes; these assessments may be performed by study investigators or by a member of the rheumatology care team. All other data collection is via questionnaires completed directly by the participants during the baseline visit or completed by participants remotely. Data on hospital appointments attended between the baseline and month 24 study visits will be captured at the month 24 visit and do not need reporting at the time of the visit. As standard care and PIFU follow-up pathways may vary depending on Trust specific guidelines, participants will be provided with site-specific instructions on how to contact their care team if required. This may be their rheumatology clinical care team or the MDT at the hospital or secondary care site. Therefore, patients allocated to the standard care arm should attend any routine appointment during the study period and patients allocated to the PIFU arm should attend any PIFU appointments required during the study period. Any routine care assessment or tests that are determined to be required during any clinic appointment should be performed as medically indicated. These appointments (outside of baseline and month 24 visits) are not study visits (Fig. 1).

Table 2 shows the scheduled assessments/activities by timepoint for the study.

**Table 2:** Scheduled assessments/activities

Timepoint	Baseline visit	Post randomisation						
		**1 week**	**6–8 weeks**	**6 months**	**11 months**	**12 months**	**18 months**	**24 months**
**Visit type/method of data collection**	**Clinic Visit**	**Remote data collection**	**Medical notes review**	**Remote data collection**	**Medical notes review**	**Remote data collection**	**Remote data collection**	**Clinic Visit**
**Assessment**								
Consent*	X							
Eligibility Assessment*	X							
Disease & treatment history*	X							
Arthritis type-specific disease activity (CDAI & DAS28-CRP or ASDAS-CRP or DAPSA) including examination of joints by blinded assessor and CRP level results from a blood test **	X							X
Demographics*	X							
MSK-HQ questionnaire*	X			X		X	X	X
EQ-5D-5L questionnaire including EQ-VAS*	X					X		X
PHQ-4 questionnaire*	X							X
PEPPI questionnaire*	X							X
Health resource use and costs questionnaires*	X			X		X	X	X
CollaboRATE & SDMQ-9 questionnaires; time spent accessing patient education material*		X						
Flare questionnaire*				X		X	X	X
Consumer health activation index (CHAI)*	X							
Single Item Literacy (SILS)*	X							
Randomisation*	X							
Training to complete remote questionnaires*	X							
Education on PIFU**	X - PIFU arm only							
Medical records review for hospital resource use*	X							X
Medical records check that patient has been booked to correct intervention*			X		X			
Medical record review intervention adherence*								X
Medical records review for flare data*								X

*Research specific activities/procedures

**Routine clinical care activities. Joint exam and blood samples for CRP level results will be done as part of routine clinical care to determine arthritis type-specific disease activity; data will be extracted from medical notes and entered into CRF

*Italicised* assessments denote completed directly by participants

### Baseline (clinic visit)

The baseline visit is a routine care visit with additional study procedures.

Once informed consent for study participation has been obtained and eligibility confirmed, baseline data will be collected/recorded by the site research team in accordance with the data collection schedule.

A physical examination of the joints will be conducted and a routine clinical C-Reactive Protein (CRP) test will be performed to calculate the specific disease activity score. Both the physical exam of the joints and CRP test will be done as part of routine care and the resulting data will be extracted from the medical notes to the study CRFs.

Participants will be asked to complete the baseline questionnaires prior to randomisation. Usually these will be completed on a study tablet-device, but in case of problems, paper copies should be completed and subsequently entered onto REDCap by the site research team. The Consumer Health Activation Index (CHAI) and Single Item Literacy Screener (SILS) will be administered verbally to the participant by the site research team prior to randomisation and then entered into the study REDCap data collection system.

Participants will then be randomised, with those allocated to the PIFU arm being provided with verbal and written information on PIFU and how to access advice if needed.

Participants will be asked whether they wish to complete follow-up questionnaires electronically via a link sent by email, via a link sent by SMS, on paper with postal return, or during a telephone call with the central study team. At, or following the baseline visit, data on hospital resource use from 12 months prior to baseline visit to date will be extracted from the participant’s medical notes and entered into REDCap by a member of the site research team.

### 1 week post randomisation (remote data collection only)

All participants will be asked to complete questionnaires about shared decision-making discussions around their suitability for PIFU using the CollaboRATE & 9-item Shared Decision Making Questionnaire (SDMQ-9) questionnaires and time spent accessing patient education materials.

### 6, 12, and 18 months post randomisation (remote data collection only)

Participants will be asked to complete the questionnaires specified in Table 2 remotely.

### 24 months post randomisation (clinic visit)

Participants will attend a clinic visit. The 24-month visit is a routine care visit with additional data collection for the study. Participants will be asked to complete the questionnaires as specified in Table 2 remotely. If the questionnaires have not been completed prior to the clinic visit, questionnaires are to be completed during the visit.

A physical examination of the joints will be conducted and a blood sample will be collected and sent to the local site laboratory for testing for CRP level to calculate the specific disease activity score. Both of these assessments are done as part of routine care and the resulting data will be extracted from the medical notes to the study CRFs. Data on disease status, flares, hospital resource use and intervention adherence (change from PIFU to standard care or vice versa) will be extracted from the participant’s medical notes and entered into REDCap.

### Study questionnaires

Questionnaires at follow-up time points will be completed remotely. Participants will select how they would like to complete study questionnaires at the time of consent — email link, text message (SMS) link, post or telephone. For those opting to complete electronically, a link will be sent to a participant either by email or SMS to a questionnaire which is unique to a participant and their timepoint/questionnaire in the study.

Where participants opt to complete paper-based questionnaires, these will be returned to the CTU by post using the prepaid envelope provided and data will be entered into REDCap by the central CTU study team. Data collected during telephone calls with the participant will be entered directly into REDCap by the central study team.

Where required, participants will be sent up to two reminders via their preferred method of questionnaire delivery. Due to the primary endpoint being collected via questionnaire, if the questionnaires are not completed after two reminders have been sent, a further final request to complete will be made by a different method via telephone or post. The questionnaires required at 24 months may be completed during the 24 month study visit if not completed prior to the visit.

For questionnaires completed remotely, if not all questionnaires have been completed the CTU study team will contact the participant to request completion of the remaining questionnaires. No more than 1 further reminder to complete the remaining questionnaires will be sent. (Note: if during the study, a participant wishes to change how they are contacted regarding the questionnaires, this is possible and should be recorded in REDCap).

### Arthritis type-specific disease activity score

The disease activity score corresponding to the type of arthritis the participants have is to be completed as follows:

- Rheumatoid Arthritis (RA): Clinical disease activity index (CDAI) and disease activity score (DAS28-CRP)
- Axial spondyloarthritis (axSpA): Axial spondyloarthritis disease activity score (ASDAS-CRP)
- Psoriatic Arthritis (PsA) & undifferentiated arthritis: Disease activity in psoriatic arthritis (DAPSA)

If a site’s usual practice is not to repeat the CRP due to a recent test result being available, or there being no medical need to repeat (i.e. arthritis appears stable) then a further CRP result is not required for the purpose of the study.

The joint examination component is to be completed by a blinded assessor whenever possible. The blinded assessor may be a suitably trained member of the clinical or research study team.

### Qualitative sub study

A qualitative sub study will collect qualitative data from three groups — patients with inflammatory arthritis who decline to participate in the TaILOR study, patients with inflammatory arthritis who participate in the TaILOR study and who are randomised to PIFU; and NHS professionals involved in their care (clinicians, nurses, research nurses, allied health professionals, managers, administrative staff). At the site feasibility stage, we will perform a survey of all sites to collect information about their hospital and the local implementation of PIFU. This will be used to support the sampling for the qualitative studies.

### Surveys of those who decline to join the study

Those who choose not to participate in the TaILOR study will be invited to complete an anonymous, short survey about why they chose not to take part. The survey comprises a mix of closed and open-ended questions exploring reasons for declining to take part in TaILOR specifically and/or research more generally. These patients will be given an invitation letter by the site staff at the time they decline to participate. The invitation letter explains why we are asking them to complete the survey and will contain a QR code to a short survey that they can choose to complete electronically. If requested, the site can provide a paper copy of the questionnaire for the participant to complete. No further contact will be made about completion of the optional survey.

### Participant interviews

Interview topic guides for all groups will be designed in collaboration with patient research partners and the study team. All who accept the invitation to take part will be offered the choice of an online or telephone interview. For the participant qualitative interviews, interpreters will be provided if required, so that participants can be interviewed in the language of their choice. Alternatively, if the participant prefers a family member or friend can act as interpreter. All interviews will take place over Teams or telephone and be audio-recorded, transcribed and de-identified. Data will be transcribed by a General Data Protection Regulation (GDPR) compliant, University of the West of England (UWE) approved, third party supplier with a data processing agreement in place. The de-identified transcripts will be kept for 4 years and then destroyed.

We will develop a purposive sampling framework and invite patients to take part in up to two qualitative interviews. Detailed baseline data collected for the study including randomisation allocation will support the purposive sampling framework. We will aim to include patients from at least 10 different sites with a mix of age, diagnosis, disease duration, gender, ethnicity, education, income, work status, health literacy and patient activation (as defined by participant SILS and CHAI questionnaire scores). We anticipate collecting data from approximately 45 interviews from approximately 30 patients in total but will continue with interviews until data saturation is met to ensure that we identify themes from a varied sample. This large sample size reflects the diverse sample that we aim to include.

Participant data for those who take part in the qualitative portion of the study will be identified using their TaILOR identification code held in a database at UWE which contains no identifiable information. A linkage document used to identify participants will be stored at UWE in a separate, password-protected document. This will only be accessed by the qualitative team at UWE.

The interview(s) will take place between 2 and 12 weeks and/or 16–24 months after randomisation with each interview lasting between 30 and 60 min. Where possible and willing, the participants will be interviewed at both time points but if insufficient participants are willing to take part in both interviews, participants may be interviewed at just one of these time points. It is not anticipated that participants will find the questions upsetting. However, participants can stop or pause the interview at any time if they are uncomfortable. Additionally, the PIS provides contact information of disease specific charities if further support/information is required. The qualitative research team have undertaken Good Clinical Practice (GCP) training and understand the need to be responsive to participants’ needs. In the interviews, we will explore patients’ views and experiences of:

- Being invited to move to PIFU, including shared decision-making
- Acceptability of PIFU and facilitators to implementation
- Any consequences and perceived negative impacts of the approach
- Individual and system level barriers and facilitators to accessing timely, safe and helpful care in the PIFU system
- Self-management and the impact of PIFU on their health

Optional consent to be invited to a maximum of two qualitative interviews will be collected as part of the consent form to participate in the TaILOR Study. The TaILOR qualitative research team will then contact participants directly with further information about the interviews, obtain consent to be interviewed prior to the first interview and arrange and carry out the interviews. Site staff and central CTU team will not routinely be informed as to whether a participant has taken part in a qualitative interview.

### Health professional/service provider interviews

We will invite approximately 25 health professionals and service providers to take part in qualitative interviews from 12 to 15 of the 32 sites participating in the main study. Health professionals (clinicians, nurses, research nurses, allied health professionals) and service providers (managerial and administrative team such as clinic booking staff, departmental and operational managers) are eligible to take part in the qualitative interviews provided they are willing to give consent. Sites will be selected to include a mix of rheumatology departments in larger teaching hospitals and smaller district hospitals, serving urban and rural communities, a range of socio-economic groups (based in the English indices of deprivation 2019 data) and different geographical areas. Across sites, we will sample for variation of individuals in profession/role and years working in rheumatology.

In the interviews, we will explore health professionals/service providers’ views and experiences of:

- The benefits and challenges of implementing PIFU in their service/at their site.
- PIFU education and potential ongoing unmet needs in terms of materials.
- Inviting patients to move to PIFU, including their perceptions of which patients are most and least likely to benefit and why.

Site staff involved with the TaILOR study will be made aware of the option to participate in an interview during the Site Initiation Visit. If the site is selected to be part of this aspect of the study, and once the site has been opened to recruitment for a minimum of 12 months, the site team will be approached to ask if there are 1 or 2 members of the team who are willing to consent to be interviewed. Further information on the interviews will be provided and those willing to be interviewed will contact the TaILOR qualitative research team directly to inform them. The TaILOR qualitative research team will then contact those health professionals/service providers selected for interview and provide the PIS and consent form. Participants will be asked to complete and return the consent form to the qualitative team ahead of the interview.

Consent will be obtained prior to starting the interview and before any data collection. Interviews should take between 20 and 40 min and participants can stop or pause the interview at any point if they become uncomfortable.

### Sample size {19}

Three hundred and fifty participants providing data on the MSK-HQ score measured at 24 months post randomisation (175 per group) will provide 90% power to detect a difference in means of minimally clinically important difference (MCID) of 4 points [18], assuming a standard deviation of 11.5 points, using a two group t-test with a 5% (2-sided) significance level. This has been inflated to 438 (219 per arm) to allow for 20% attrition and crossover.

The MCID, estimated using the standard error as no anchor-based estimates were available [18], and the standard deviation of 11.5 points is estimated using Healthcare Quality Improvement Partnership (HQIP) National Audit for Early Inflammatory Arthritis Audit data [19]. There are no similar studies on which to base the estimated attrition rate, 20% is chosen to be conservative and in line with the wider RCT literature.

### Recruitment {20}

We aim to recruit 438 participants from a minimum of 30 rheumatology centres within NHS hospitals/care sites in the UK. Assuming an average NHS Trust serves a population of 300,000 adults, we will have a recruitment pool of 9 million adults. Of these, 1.5% people have inflammatory arthritis and NHSE figures from existing PIFU centres estimate that 40% may be eligible for PIFU, giving an estimate pool of 54,000 potentially eligible patients across 30 sites.

The anticipated monthly recruitment rate is 49 participants per month for 9 months. This equates to 2 patients per site, per month, taking into account staggered site opening. It is expected that all sites will be open within 5 months of starting recruitment.

The following methods will be used by the usual care team to identify potentially eligible participants:

- Identification during routine clinic visits
- Searching of clinic records/hospital databases to identify individuals that may be eligible to be approached about the study

Potentially eligible patients may be provided with a PIS, where appropriate by a member of their usual care team. Eligibility for the study will be confirmed by the patient’s treating clinician.

## Assignment of interventions: randomisation

### Sequence generation: who will generate the sequence {21a}

Randomisation will be performed using a minimisation algorithm generated using REDCap. Participants will be randomised via a centralised validated computer randomisation program through a secure (encrypted) web-based service, provided by the Oxford Clinical Trials Research Unit (OCTRU), accessed via the REDCap study database.

The constraints for the randomisation schedule will be determined by the Oxford Clinical Trials Research Unit (OCTRU) study statistician and full details will be detailed in the randomisation and blinding plan.

### Sequence generation: type of randomisation {21b}

Consented participants will be randomly allocated (1:1) to one of the study arms. Randomisation will be performed using a minimisation algorithm to ensure balance between the two arms using stratification factors:

- Participant disease presentation (either predominantly axial, or predominantly peripheral involvement according to treating clinician)
- Recruiting site

Stratification by participant diagnosis will ensure that the arms are balanced in terms of their pattern of disease, as these are associated with different prognoses. Stratification by recruiting site will ensure that the treatments are balanced according to the different demographics of the cohort seen at different centres and according to the difference in practice associated at a local level for the standard care (comparator) arm.

Classification of participants as axial or peripheral will be done by their treating clinician based on their clinical assessment.

### Allocation concealment mechanism {22}

The minimisation will be implemented using an online system with a random element to prevent predictability of the next allocation. The first few participants will be randomised using simple randomisation, to seed the minimisation algorithm, and a non-deterministic probabilistic element will be included to prevent predictability of treatment allocation.

### Implementation {23}

Randomisation will be initiated by the site team via an automated, secure (encrypted), web-based randomisation module incorporated into the study-specific REDCap data collection system instance provided by OCTRU.

## Assignment of interventions: blinding

### Who will be blinded {24a}

Only the person undertaking the disease activity score assessment will be blinded to study arm (where this is possible). Neither the participants nor any other staff involved needs to be blinded. Table 3 provides an overview of the blinding status of all individuals involved in the conduct and management of the study. A blinded analysis will be carried out to look into the distribution of variables, missing data distributions, and outliers.

**Table 3:** Blinding

Role in study	Blinding status	Additional information
Participants	Not blinded	It is not possible to blind due to nature of the intervention. Participants will be told their treatment allocation after randomisation
Site research staff including the Principal Investigator (PI) but excluding disease activity assessors	Not blinded	Not possible due to the nature of the intervention
Disease activity assessor	Blinded whenever possible	Arthritis type-specific disease activity should be assessed by an assessor who is blinded to the intervention allocation wherever possible. Assessment by a non-blinded assessor will not be considered a protocol deviation
Chief Investigator (CI)	Not blinded	It is not possible to blind the CI as they may be the primary clinician for those participants recruited at their site, however they will be blinded to allocations for participants at other sites
Data collection system programmer	Not blinded	The programmer is responsible for the management of the randomisation system and the REDCap data collection system and will have access to all unblinded datasets within both systems
TaILOR Study Management staff within OCTRU	Not blinded	Study Management staff within EMR group will not be blinded to intervention allocations as site staff may require support for randomisation or other aspects of the study, or participants may contact the study team directly Study Management staff will also have access to the unblinded datasets within the study randomisation system and REDCap to ensure data quality and undertake central monitoring activities
Data Management	Not blinded	Data management staff will have access to the unblinded datasets within the study randomisation system and REDCap to ensure data quality and undertake central monitoring activities
Study Statistician and Senior Study Statistician	Not blinded	The study statistician and senior study statisticians will have access to treatment allocations, as required, to generate the Data Monitoring Committee (DMC) closed reports and the final analysis
Health Economist	Not blinded	The Health Economists will have access to treatment allocations, as required, to generate DMC closed reports and the final analysis
Qualitative Researchers	Not blinded	The qualitative researchers will have access to treatment allocations to allow them to invite PIFU allocated patients for qualitative interviews

### How will be blinding be achieved {24b}

Given the nature of the intervention, it was not possible to design this as a double-blinded trial.

A disease assessor is considered blinded if they are not aware of the intervention allocation of the participant. No study-specific processes are defined by the protocol to achieve blinding of disease assessors. If the disease assessor was not blinded, this is recorded within the study database.

### Procedure for unblinding if needed {24c}

NA — the only blinding in the study is the disease activity assessor (though not required). There is no need for an unblinding procedure for this study.

## Data collection and management

### Plans for assessment and collection of outcomes {25a}

The data management aspects of the quantitative study are summarised in the Data Management section with details fully described in the study-specific Data Management Plan. Collection and storage of data will be managed in accordance with OCTRU Standard Operating Procedures (SOPs). University of the West of England will manage the qualitative sub-study (acting as a joint data controller) and will be responsible for the collection, storage and destruction of personal data relating to the qualitative sub-study in compliance with UWE SOPs and processes. The only aspects of the qualitative study which will be managed in accordance with OCTRU SOPs are the sharing of data of participants who consented to be contacted for an interview and archiving of study results.

### Plans to promote participant retention and complete follow-up {25b}

As the study is set within standard care, participants are likely to remain under the same rheumatology team regardless of intervention, leading to minimal disruption to ongoing follow-up care. Additionally, the central study team will send remote questionnaires and follow-up at set intervals. Finally, the patient and public contributors are planning videos that can be shared with participants during the follow-up period to enhance engagement.

### Data management {26}

Data will, wherever possible, be collected in electronic format with direct entry into REDCap by site staff or participants. Electronic data collection has the major advantage of building “data logic” into forms, minimising missing data, data input errors and ensuring the completeness of consent and assent forms. REDCap is a secure, web-based application designed to support data capture for research studies, providing (1) an intuitive interface for validated data entry; (2) audit trails for tracking data manipulation and export procedures; (3) automated export procedures for seamless data downloads to common statistical packages; and (4) procedures for importing data from external sources.

All data entered will be encrypted in transit between the client and server. All electronic patient-identifiable information, including electronic consent forms, will be held on a server located in an access-controlled server room at the University of Oxford. The data collection system and server are backed up to a secure location on a regular basis. Details of the data collected, where it is stored and who has access to it along with a fair processing statement will be available for the participants within the study participant information sheet. Direct access to source data/documents will be required for study-related monitoring and/or audit by the Sponsor, research team or NHS Trust or regulatory authorities as required.

Data captured during phone calls to participants or from paper-based study questionnaires returned to the study office will be entered into REDCap by suitably trained central study office staff. Full details of this process will be recorded in the Data Management Plan. Identifiable data will only be accessible by members of the research team with a demonstrated need (managed via access controls within the application) and only used to communicate with the participant (e.g. for sending follow-up reminders for online form completion or telephone follow-up).

## Confidentiality {33}

### Collection and use of personal identifiable information

Contact details email address, postal addresses and phone number (mobile and/or home phone) of study participants will be collected at the time of consent for the following purposes, and where an activity is optional, only with the specific consent of the participant:

- Requesting completion of follow-up questionnaires
- Reminders to complete follow-up questionnaires
- Sending a copy of the completed consent form by email (for any participants that consent electronically and wish to receive a copy by email)
- Sending an invitation for qualitative research interviews
- Sending the summary of the results of the study

The PIS explains what contact details will be collected and how these will be used.

Sharing of contact details with the TaILOR qualitative research team at UWE will be via secure methods in compliance with OCTRU SOPs. A data sharing agreement is in place to cover these activities.

Site staff at participating sites will ensure that contact details for study participants are up to date when participants attend for study visits. NHS numbers will be collected from participants living in England to enable additional health economics and/or outcome data to be obtained from NHSE via OpenSafely. 

#### Use of audio/visual recording devices

Qualitative interviews will be audio recorded to enable transcription and analysis. Transcriptions will be de-identified. Audio recordings will be deleted once the transcription process is complete. The use of audio recording devices will be under the strict control of UWE SOPs and policies.

#### Storage and use of personal data

During the study personal data will be stored and used in accordance with the OCTRU SOP. This ensures that all personal data collected during the study is recorded, handled and stored in accordance with the requirements of the UK GDPR.

All electronic participant-identifiable information will be held on a secure, password-protected database accessible only to authorised personnel. Paper forms with patient-identifiable information will be held in secure, locked filing cabinets within a restricted area. The processing of the personal data of participants will be minimised wherever possible by the use of a unique participant study number on study documents and any electronic systems. Personal data on all documents will be regarded as confidential. The study staff will safeguard the privacy of participant’s personal data. The use of all personal data in the study will be documented in a study-specific data management plan which details what and where personal data will be held, who will have access to the data, when personal data will be anonymised and how and when it will be deleted. The Investigator site will maintain the patient’s anonymity in all communications and reports related to the research.

Any data breach will be highlighted to the relevant site staff and reported as required by the UK GDPR and Data Protection Act 2018. This will also be deemed a protocol deviation.

#### Access to participants’ personal identifiable data during the study

Access to participants personal identifiable data will be restricted to individuals authorised to have access. This includes (a) members of the research team at participating study sites with delegated responsibility by the site PI and (b) members of the central CTU study team involved in the conduct/management of the study where this is necessary for their role. Research staff that are not part of the potential participant’s direct healthcare team will not have access to personal identifiable data until the individual has given their consent to take part in the study or the participant has indicated to their direct healthcare team that they wish to be contacted by a member of the site research team — permission for this will be recorded in the individual’s medical notes. The PIS clearly describes who will have access to the participants personal identifiable data during the study and explicit consent is obtained from study participants for such access, including participants who consent to the qualitative sub-study.

#### Destruction of personal identifiable data

Personal identifiable data will be destroyed as soon as it is no longer required — the time point for this destruction is detailed in the study data management plan and is in accordance with OCTRU SOPs which comply with the UK GDPR. Personal identifiable data collected and used as part of the qualitative sub-study will be destroyed within one year of the end of the study in accordance with UWE specific policies and SOPs.

Personal identifiable data may be retained longer than the duration of study in accordance with the University of Oxford’s minimum mandatory archiving period.

#### Participant identification log

The site research team must keep a separate log of enrolled patients’ personal identification details as necessary to enable them to be tracked. These documents must be retained securely, in strict confidence. They form part of the Investigator Site File (ISF) and are not to be released externally.

## Statistical methods

### Statistical methods for primary and secondary outcomes {27a}

The statistical aspects of the study are summarised here with details fully described in a separate SAP that will be drafted early in the study and finalised prior to the final analysis data lock. The SAP will be written by the Study Statistician in accordance with the current OCTRU SOPs. The TSC and DMC will review and, if necessary, provide input into the SAP.

The statistical analyses will be performed once the 24-month follow-up has been reached by the last participant and sufficient time has been allowed for data cleaning.

Reporting of results will be in accordance with the CONSORT Statement and relevant extensions [20, 21].

Intention-to-treat will be the main analysis strategy and will be adopted for the primary and all secondary outcomes. In other words, the analysis will principally be focused on a treatment policy estimand.

It is anticipated that all statistical analysis will be undertaken using Stata [22] or other validated statistical software tools.

The primary outcome (MSK-HQ at 24 months) will be compared using a mixed-effects linear regression model with repeated measures (level 1), grouped within participants (level 2). Models will include treatment, baseline MSK-HQ score, time as a dummy variable and time-by-treatment interaction incorporated as fixed effects. The model will include outcome data at 6, 12, 18 and 24 months. As recommended by International Council for Harmonisation (ICH) E9, the model will also adjust for the minimisation factors: diagnosis (axial, peripheral) as a fixed effect and recruiting site as a random effect [23]. Treatment effects for MSK-HQ at each follow-up time point (6, 12 and 18 months) will be estimated from the same model.

A sensitivity analysis will also be undertaken that will further incorporate other important prognostic variables, such as type of arthritis. Results will be displayed as estimates and 95% confidence intervals. Heterogeneity of the treatment effect across important subgroups: diagnosis (axial, peripheral), recruiting site, and other important prognostic factors will be presented exploratively using forest plots. Secondary analysis of MSK-HQ over 24 months will be analysed using the area under the curve (AUC) summary statistics and a model with no time interaction to estimate the overall effect of the intervention over all time points.

Continuous secondary outcomes measured over time (EuroQol 5 Dimensions 5 Level Version (EQ-5D-5L) and EuroQol Visual Analogue Scale (EQ-VAS) will be compared using methods in line with the primary analysis. Continuous secondary outcomes measured at baseline and one other fixed timepoint (Patient Health Questionnaire-4 (PHQ-4), Perceived Efficacy in Patient-Physician Interactions (PEPPI), DAS28-CRP, ASDAS-CRP, DAPSA) will also be compared using similar methods to the primary, but without repeated measures effects. Binary outcomes (progression to biologic therapies) will be compared using a mixed effects logistic regression model, adjusted for treatment group and minimisation factors. Comorbidities will be summarised by frequencies and percentages.

All treatment comparisons will be reported with 95% confidence intervals and a two-sided significance level of 5% will be used to test statistical significance.

### Who will be included in each analysis {27b}

The principal analysis will be performed on the intention-to-treat population, analysing participants with available outcome data in their randomised groups, regardless of adherence. Intention-to-treat (ITT) will be the main analysis strategy and will be adopted for the primary and all secondary outcomes. In other words, the analysis will primarily focus on the treatment policy estimand. The principal stratum strategy will be used as a supplementary analysis.

### How missing data will be handled in the analysis {27c}

Sensitivity analyses will explore the impact of missing data. The procedure for handling spurious or missing data will be described in the SAP. The study will attempt to collect data as completely as possible. Missing data, for example due to withdrawal, protocol deviation or patient loss to follow-up, will be summarised and patterns analysed. Analysis of the primary and all secondary outcomes will be performed using available data. If there is sufficient or differential missing data, sensitivity analyses using multiple imputation techniques will be performed. These will explore the possibility of data being missing at random as well as departures from this assumption.

### Methods for additional analyses (e.g. subgroup analyses) {27d}

Potential risk factors, and their association with optimal PIFU outcomes, will be explored using simple summary statistics. Optimal PIFU outcomes include musculoskeletal quality of life (total MSK-HQ) and the individual understanding of your condition and confidence in self-management questions (taken from MSK-HQ). Categorical data will be summarised by counts and percentages. Continuous data will be summarised by mean, standard deviations and range if data is normally distributed. Median, interquartile range and range will be reported if data is skewed. No imputation for missing data will be performed. No formal statistical testing will be undertaken. All analyses will be exploratory and not constitute univariable screening.

### Qualitative analysis

Both the patient and the clinician/service provider data sets will be analysed using a hybrid thematic analysis, integrating deductive and inductive coding [24, 25]. Thematic analysis involves the identification of themes and patterning within the transcript data to describe and illuminate the phenomenon under research. The deductive, theory-driven coding will be informed by the multifaceted theoretical framework of acceptability (TFA) [26] which can be used to understand acceptability from the perspective of both the providers and recipients of an intervention. The TFA comprises seven component constructs: affective attitude, burden, perceived effectiveness, ethicality, intervention coherence, opportunity costs, and self-efficacy [26]. The inductive, data-driven coding will enable us to identify further insights from participants about PIFU in clinical practice that are not within the scope of the TFA.

The inclusion of a substantial qualitative component in our study design ensures that the delivery and evaluation of PIFU will be informed by patients’ and health professionals /service providers’ views and experiences. It will also enable us to identify variation between patients and health professionals/service providers and across sites. These detailed insights will help us to understand how best to tailor PIFU at both an individual and system level. These insights will be further developed by including our qualitative analysis in a mixed methods process evaluation.

### Mixed method process evaluation

A mixed method process evaluation will be utilised to triangulate the qualitative and quantitative findings. Samples, patterns and interpretation/findings between the quantitative and qualitative data sets will be compared to deepen the understanding of context, mechanisms, outcomes and implementation of PIFU across sites.

A joint analysis plan will be written that will set out the key functions of our mixed methods process evaluation, informed by the Medical Research Council (MRC) guidance [27]. The joint analysis plan will describe the processes to be studied, the methods to be used and how process and outcome data will be integrated when analysing the results.

### Health economics

A within-trial economic evaluation will estimate the incremental cost and cost-effectiveness of PIFU compared with standard care from an NHS perspective. The base case analysis will take a 24-month time horizon. Data on NHS resource use over the 12 months before randomisation and the two-year study period will be obtained from participant questionnaires and hospital medical records. A sensitivity analysis will take a societal perspective and will also include costs incurred by patients, their families and employers. This will use data from patient-reported questionnaires on the cost of travelling to outpatient visits and time off work due to inflammatory arthritis.

To maximise statistical power and minimise patient burden, the analysis will focus on the medications, procedures, investigations and admissions that are most likely to drive the NHS cost difference between PIFU and standard care. Costing analyses will include the following resources: all outpatient consultations; all interactions with primary care professionals; accident and emergency consultations; DMARD medications for inflammatory arthritis; rheumatology advice lines; and procedures, investigations and admissions related to inflammatory arthritis disease process or treatments. The procedures, investigations and admissions related to inflammatory arthritis and treatment were prespecified in discussions between health economists and clinicians. This includes: relevant imaging such as musculoskeletal ultrasound; joint replacement; injection of intra-articular medications; and admissions for infection. Data on medications other than DMARDs will not be collected as they are not expected to have a material impact on incremental costs.

EQ-5D-5L will be used to estimate Quality Adjusted Life Years (QALYs); if no validated UK tariff is available, we will use the cross-walk tariff [28]. Cost-effectiveness will be expressed as incremental cost per QALY. Bootstrapping will be used to quantify uncertainty around costs, QALYs and cost-effectiveness ratios as 95% confidence intervals and cost-effectiveness acceptability curves. Analyses will adjust for covariates including demographics, costs in the 12 months before randomisation and baseline EQ-5D-5L. The economic evaluation will follow the National Institute for Health and Care Excellence (NICE) reference case [29] and Consolidated Health Economic Evaluation Reporting Standards (CHEERS) checklist [30].

Subgroup analyses will be conducted to see how costs, QALYs and cost-effectiveness vary between patient groups with different baseline characteristics. Sensitivity analyses will assess whether the conclusions are sensitive to changes in the methods and assumptions. A secondary analysis will extrapolate beyond the two-year time horizon, assuming that 24-month utilities will remain constant beyond month 24. This will assume biennial costs beyond month 24 will equal the costs in the trial other than any DMARD medication changes initiated at or by the 24-month visit. A further secondary analysis will reweight trial participants to generalise trial results to reflect the distribution of baseline characteristics in the wider population using data from OpenSAFELY. A distributional cost-effectiveness analysis will be performed to examine how incremental costs and QALYs of PIFU vary between income quintiles and assess the distributional implications of the policy. A health economics analysis plan (HEAP) will be prepared before data collection is completed.

### Interim analyses {28b}

The main outcomes will be analysed as stated in the analysis plan once the study follow-up has been completed. No formal interim analyses of treatment effect are planned for any of the study outcomes.

As no formal interim analyses are planned, no stopping rules have been incorporated into the study design. An independent DMC met early in the trial to agree its terms of reference. They will review the confidential accumulating data at regular intervals and can recommend pausing or stopping the study in the event of safety concerns.

An internal pilot with predefined stop–go criteria to assess feasibility of recruitment was reviewed after 3 months of recruitment. The internal pilot study mirrored the procedures and logistics undertaken in the main definitive study. Predefined stop–go criteria and outcome of this pilot phase are outlined in Table 4.

**Table 4:** Internal pilot criteria and progression guidance

Progression guidance	Criteria	Outcome
Continue with study — no action required	**Recruitment** = 75% + of 3 month target (68 participants) **Number of centres opened** = 20 +	**Recruitment**: 76 participants **Number of centres opened**:25 **Action**: Continue with study, no action required
Continue with study — action required: 1. Review recruitment strategies and modify/monitor closely 2. Report to TSC	**Recruitment** = 50–75% of 3 month target (45–67 participants) **Number of centres opened** = 10–20	
Stop	**Recruitment** = <50% of 3 month target (45 participants) **Number of centres opened** = <10	

The TMG closely monitored the progression criteria, and together with the TSC and DMC performed a full review at the end of the internal pilot. At the time of the review, the study met the ‘green’ progression guidance to continue with the study with no action required. Therefore, the study progressed seamlessly into the main phase. Data from the internal pilot will contribute to the final analysis.

### Protocol and statistical analysis plan {5}

The SAP and HEAP will be submitted for publication in an open-access peer-reviewed journal in accordance with the Guidelines for the Content of Statistical Analysis Plans in Clinical Trials [31] prior to any comparative analyses.

## Oversight and monitoring

### Composition of the coordinating centre and trial steering committee {3d}

The study will be coordinated by OCTRU based at the University of Oxford. A TMG will be established for the study and operate in accordance with a study-specific TMG charter. The TMG will manage the study, including the clinical and practical aspects, and will meet approximately monthly to assess progress. Other specialities/individuals will be invited as required for specific items/issues.

### Trial steering committee

The TSC, which includes independent members, provides overall supervision of the study on behalf of the funder. The TSC will act in accordance with a TSC charter which will outline its roles and responsibilities. Full details including names will be included in the TSC charter. Meetings of the TSC will take place at least once a year during the recruitment period. An outline of the remit of the TSC is to:

- Monitor and supervise the progress of the study towards its interim and overall objectives
- Review at regular intervals relevant information from other sources
- Consider the recommendations of the DMC
- Inform the funding body on the progress of the study

The TSC will consider, and act, as appropriate, upon the recommendations of the DMC

### Composition of the data monitoring committee, its role and reporting structure {28a}

An independent DMC was established for this study. The DMC will adopt a DAMOCLES-based charter, which defines its terms of reference and operation in relation to the oversight of the study. The DMC will meet regularly throughout the study at time-points agreed by the Chair of the Committee and the CI. At a minimum this will be on an annual basis. The DMC will review the patient reported flare data generated and make recommendations as to whether the protocol should be amended to protect patient safety. Recommendations of the DMC will be discussed between the CI, TSC, and the Sponsor.

### Frequency and plans for auditing trial conduct {29}

All aspects of the study conduct may be subject to internal or external quality assurance (QA) audit to ensure compliance with the protocol, GCP requirements and other applicable regulations or standards. Such audits or inspections may occur at any time during or after the completion of the study. Investigators and their host.

### Protocol amendments {31}

All amendments will be generated and managed according to the CTUs SOPs to ensure compliance with applicable regulation and other requirements. Written confirmation of all applicable Research Ethics Committee (REC), Sponsor and local approvals must be in place prior to implementation by investigators as applicable for the amendment type. The only exceptions are for changes necessary to eliminate an immediate hazard to study participants in the case of an urgent safety measure to protect study participants from any immediate hazard to their health or safety.

It is the Investigator’s responsibility to update participants (or their authorised representatives, if applicable) whenever new information becomes available that might affect the participant’s willingness to continue in the study. The Investigator must ensure this is documented in the participant’s medical notes and the participant is re-consented if appropriate.

### Dissemination policy {8}

The Sponsor will retain ownership of all data arising from the study. Publication and dissemination of study results will be in accordance with OCTRU SOPs and irrespective of study findings. The study results will be published in an open-access journal, in accordance with the NIHR’s policy on open-access research as well as appropriate conferences. The study will be reported following the Consolidated Standards of Reporting Trials guideline (CONSORT) including any applicable extensions to this. The Template for Intervention Description and Replication (TIDieR) statement will be used for reporting the intervention.

All data will be presented such that no individual participants can be identified. Publications may include direct quotations from the qualitative interviews.

A summary of the study results for study participants will be written collaboratively with clinicians and patient representatives and distributed to study participants by the Central CTU Study Office, unless an individual has stated they do not wish to receive such a summary. The PIS will also include a link to the study website where participants will be advised that the results will be published. Findings will also be available via the CTU website and social media. Newsletters, social media, and relevant patient-facing charities will also be used to ensure the results of the study are communicated to the wider community once they are available.

A stakeholder engagement event is planned to discuss the results, interpretation and policy implications and both this study and the findings of the observational study with the research programme. Key stakeholders for such a meeting, with a focus on diversity in representation, would be patients, rheumatology teams, hospital managers and NHSE. The outcome of this meeting will form an additional research output from the project and we believe would be of great interest to patients, clinicians and policy makers internationally. We will also share our findings to support work in other speciality areas, primarily through our links with relevant NHS teams.

Authorship for future trial publications will be determined in accordance with the ICMJE criteria. NIHR Associate Principal Investigators will be recognised under group authorship, and professional medical writers will not be used.

## Discussion

Inflammatory arthritis comprises lifelong conditions, such as RA, AxSpA and PsA that require long-term treatment and regular monitoring of disease and treatment. The implementation of PIFU where patients request appointments as required may offer an opportunity to empower patients, supporting self-management and a sense of control over healthcare decisions. It could improve patients’ experience of care and enable clinicians to focus their attention on those whose clinical need is greatest. However, there are concerns from patients and clinicians that PIFU should not compromise patients’ short-term or long-term outcomes, nor result in increased health inequalities and that PIFU should be tailored to the individual patient and the service concerned. To date, there are small studies of PIFU in RA and none in other arthritis. The research within the TaILOR programme of work is required to provide further data on PIFU across different forms of inflammatory arthritis in the UK. This can help to configure services to optimise outcomes, reassure patients and clinicians of the effectiveness and safety of PIFU and ensure that suitable patients are selected for this pathway.

### Trial status

Protocol version 2.0 (20 January 2025) was approved on 22 January 2025. The study opened to recruitment on the 21 March 2025 and was initially expected to close by 31 December 2025. Protocol V3.0 (04 December 2025) was approved on 04 December 2025 to extend recruitment by 1 month (ending 31 January 2026).

## Supplementary Information

Supplementary Material 1.

## Data Availability

Upon completion of the study, anonymised research data may be shared with other organisations on request to the CI and in accordance with the data sharing policies of OCTRU, the Sponsor and funder(s).

## Abbreviations

ASDAS: Axial Spondyloarthritis Disease Activity Score
AUC: Area under the curve
AxSp: AAxial spondyloarthritis
BritPACT: British PsA Consortium
BSR: British Society for Rheumatology
CDAI: Clinical Disease Activity Index
CHAI: The Consumer Health Activation Index
CHEERS: Consolidated Health Economic Evaluation Reporting Standards
CI: Chief investigator
CONSORT: Consolidated Standards of Reporting Trials guideline (CONSORT
CRF: Case Report Form
CRP: C-reactive protein
CTU: Clinical trials unit
DAPSA: Disease activity in psoriatic arthritis
DAS28-CRP: Disease Activity Score (28 joint)—CRP
DMARDs: Disease-modifying anti-rheumatic drugs
DMC: Data monitoring committee
EMR: Experimental medicine and rheumatology
EQ-5D-5L: EuroQol 5 Dimensions 5 Level Version
EQ-VAS: EuroQol Visual Analogue Scale
GCP: Good clinical practice
GDPR: General data protection regulation
GRAPPA: Group for Research and Assessment of Psoriasis and Psoriatic Arthritis
HAQ: Health Assessment Questionnaire
HEAP: Health Economics Analysis Plan
HQIP: Healthcare Quality Improvement Partnership
ICF: Informed consent form
ICH: International Council for Harmonisation
ISF: Investigator Site File
ISRCTN: International Standard Randomised Controlled Trials Number
ITT: Intention to treat
MCID: Minimally clinically important differences
MDT: Multidisciplinary Team
MRC: Medical Research Council
MSK-HQ: Musculoskeletal Health Questionnaire
NASS: National Axial Spondyloarthritis Society
NDORMS: Nuffield Department of Orthopaedics, Rheumatology and Musculoskeletal Sciences
NHS: National Health Service
NHSE: National Health Service England
NICE: National Institute for Health and Care Excellence
NIHR: National Institute for Health and Care Research
NRAS: National Rheumatoid Arthritis Society
OCTRU: Oxford Clinical Trials Research Unit
OMERACT: Advancing Outcome Measurement in Rheumatology
OPEN ARMS: Oxford Patient Engagement Network for Arthritis and Musculoskeletal Conditions
PAPAA: Psoriasis and Psoriatic Arthritis Alliance
PEPPI: Perceived Efficacy in Patient-Physician Interactions
PHQ-4: Patient Health Questionnaire-4
PI: Principal investigator
PIFU: Patient initiated follow up
PIS: Patient information sheet
PPI: Patient and public involvement
PROs: Patient reported outcomes
PsA: Psoriatic arthritis
QA: Quality assurance
QALY: Quality adjusted life year
QoL: Quality of life
RA: Rheumatoid arthritis
REC: Research Ethics Committee
REDCap: Research Electronic Data Capture
SAP: Statistical analysis plan
SDMQ-9: 9-Item Shared Decision Making Questionnaire
SILS: Single item literacy screener
SOP: Standard operating procedure
TFA: Theoretical framework of acceptability
TIDieR: Template for Intervention Description and Replication (TIDieR)
TMG: Trial Management Group
TSC: Trial Steering Committee
UK: United Kingdom
UWE: University of the West of England

## Footnotes

1 Self-reported by patient. No pregnancy testing is required
