# Supplementary material for: TaILOR: a randomised trial to compare the clinical and cost-effectiveness of a patient-initiated follow-up (PIFU) strategy compared to standard care pathways in people with inflammatory arthritis: a study protocol
Source: Trials. 2026 Jul 4;27:475. doi: 10.1186/s13063-026-09868-0 (PMC13343589; doi:10.1186/s13063-026-09868-0)
Supplement: Supplementary file 1 — Supplementary Material 1. [file 13063_2026_9868_MOESM1_ESM.docx]

Supplementary Information

**Equality, Diversity and Inclusion**

We have considered a wide variety of underserved groups that may need additional support in order to take part in the study. This includes patients from different ethnic backgrounds, those with lower educational levels or health literacy, people who are socially disadvantaged, from remote communities, have language barriers, or may be impacted by digital exclusion.

In order to support equality, diversity and inclusion in the study population, the following have been incorporated into the study design and will be undertaken during the study:

- The study will be run at a large number of centres to include both rural and urban settings as well as centres with high levels of ethnically-diverse populations.
- Throughout the study design, we have tried to minimise the burden on participants in terms of research visits (reducing costs for travel and time away from work/caring responsibilities) by embedding these into planned clinic appointments. Where practical, we have included remote follow-up with reporting of PROs and resource use. These outcomes in the study will be collected electronically for the majority of the participants. However, we have included support for people to use either paper or telephone methods of data collection if they prefer to reduce digital exclusion and support those with lower educational levels or health literacy.
- Demographic data (including ethnicity) will be collected as part of screening data to monitoring inclusivity. This data will be monitoring by the Trial Management Group (TMG) and will be used to assess the representativeness of the study population and to allow additional interventions at key sites if patients from particular groups are declining involvement in the study.
- We will ensure that appropriate materials are in place at all sites to support patients with accessing and understanding PIFU within their rheumatology team including support about when to contact the team. This will include a video (provided by the BSR) which has been developed to ensure it is representative of the diversity that exists in the UK and is available with subtitles for translation.
- The PIS includes a 1-page pictorial summary of the study to support the initial approach of patients.
- The PIS will be translated into 5 commonly spoken non-English languages to support the recruitment of those for whom English is not their first language. Sites were asked what languages will be useful for recruitment locally as part of the site feasibility questionnaire. The five languages selected were Polish, Urdu and Punjabi, Mandarin and Hindi.
- Study meetings will be held to focus on EDI during the planning stage and initial recruitment period of the study. This will include our Patient and Public Involvement (PPI) members, PPI co-ordinator and wider representation from the arthritis community (recruited via the confirmed charity partners) to support development of study resources and approaches as well as develop suggestions for sites.
- We will support sites to encourage patients who do not regularly attend appointments, and thus may need additional support to navigate PIFU, to be approached about the study, as they may be a group who are ideally suited to the study intervention PIFU
- A budget of up to £500 per site is available to support them to implement locally relevant activities to increase the diversity of participants approached about the study.
